# Nutrition Intervention and Microbiome Modulation in the Management of Breast Cancer

**DOI:** 10.3390/nu16162644

**Published:** 2024-08-10

**Authors:** Yue Jiang, Yuanyuan Li

**Affiliations:** Department of Nutrition and Food Science, University of Maryland, College Park, MD 20742, USA; yjiang11@umd.edu

**Keywords:** breast cancer, gut microbiome, nutrition intervention, bioactive compound

## Abstract

Breast cancer (BC) is one of the most common cancers worldwide and a leading cause of cancer-related deaths among women. The escalating incidence of BC underscores the necessity of multi-level treatment. BC is a complex and heterogeneous disease involving many genetic, lifestyle, and environmental factors. Growing evidence suggests that nutrition intervention is an evolving effective prevention and treatment strategy for BC. In addition, the human microbiota, particularly the gut microbiota, is now widely recognized as a significant player contributing to health or disease status. It is also associated with the risk and development of BC. This review will focus on nutrition intervention in BC, including dietary patterns, bioactive compounds, and nutrients that affect BC prevention and therapeutic responses in both animal and human studies. Additionally, this paper examines the impacts of these nutrition interventions on modulating the composition and functionality of the gut microbiome, highlighting the microbiome-mediated mechanisms in BC. The combination treatment of nutrition factors and microbes is also discussed. Insights from this review paper emphasize the necessity of comprehensive BC management that focuses on the nutrition–microbiome axis.

## 1. Introduction

Breast cancer (BC) is one of the most common cancers globally and usually occurs among women. As Western dietary patterns prevail globally, characterized by the increased consumption of processed, sweetened, and high-fat food, the incidence of BC is rising in both developed and developing countries [1]. Other BC-related factors include early menarche, low physical activity, and delayed childbirth [1]. Clinically, BC is a complicated and heterogeneous disease that requires a multifaceted management approach. Although the incidence rate is increasing, the survival rate is also rising, attributed to increased public health awareness, early detection, and advanced cancer therapies. An increasing number of studies focusing on various dietary patterns, micronutrients, and bioactive compounds indicate the importance of nutrition intervention in improving treatment outcomes in BC patients and survivors [2].

The gut microbiome, known for its significant influence on human health, has emerged as a crucial study area in understanding BC progression. Although the exact causes of cancer have not been clarified yet, statistics show that microorganisms may be a contributor to about 15–20% of cases of cancer progression [3]. Considering the significant roles of the gut microbiome in human health, studies of the effects of the microbiome on BC progression have become vital areas [4]. Recent studies have focused on unraveling the complexities of how the diversity, composition, and functionality of microbiomes, notably the gut microbiome, affect BC treatment during different stages and response to cancer therapies, as well as prevent BC recurrence and remission. The gut microbiome is considered a double-edged sword because of its contribution to tumor-promoting inflammation and its protective effects on regulating the anticancer immune response [5]. The intricate roles of the gut microbiome encompass modulation, reabsorption, participation in enterohepatic circulation, and finally, systemic estrogen levels [6]. Notably, gut bacteria can affect estrogen levels through the secretion of β-glucuronidase. This enzyme deconjugates estrogen to its free, biologically active form. This process increases the availability of free estrogens for reabsorption, thereby contributing to the risk of developing hormone-driven malignancies like BC [7].

According to epidemiological and preclinical investigations, certain dietary nutrients, such as carbohydrates, saturated fats, and red meats, may increase the risk of BC [8]. These foods have been associated with increased circulating levels of endogenous estrogen, insulin-like growth factor (IGF)-1, and pro-inflammatory cytokines, which may contribute to BC progression [9]. Conversely, dietary nutrients like fiber, omega-3 polyunsaturated fatty acids (PUFAs), and beneficial vitamins enriched in fruits and vegetables have shown protective effects on reducing oxidative stress and chronic inflammation that potentially impede BC development and progression [10]. In addition, dietary patterns are the major factors contributing to changes in the composition and function of the gut microbiome. For example, compared to omnivores, a vegetarian diet contributes to a higher microbial diversity enriched with more beneficial bacteria such as *Bacteoridetes*, *Prevotella*, and *Roseburia* [11]. In relation to BC, healthy dietary patterns may potentially limit the activity of β-glucuronidase and reduce the plasma estrogen concentration, leading to increased estrogen secretion in the fecal sample [12]. 

Beyond the traditional treatment, there is an evolving number of new treatment strategies that focus on the interplay between nutrition intervention and microbiome modulation. Studies explore the impact of specific healthy dietary patterns, such as the Mediterranean diet, the prudent diet, and plant-based diets on inflammation, hormone levels, and overall health in BC patients [13]. Additionally, numerous studies focus on the effects of dietary bioactive compounds with anti-inflammatory and anti-carcinogenic properties on BC chemoprevention and therapy [14]. Strategies focusing on remodulating the gut microbiota, such as probiotics and prebiotics, are being explored for their potential to enhance treatment efficacy and reduce treatment-related side effects [15]. In this review, we delineate a thorough overview of the current nutrition intervention and microbiome-driven strategies for BC prevention and therapy and provide the up-to-date evidence shown in both clinical and animal trials. Evidence from these studies helps integrate this strategy into BC treatment plans and optimize BC care.

## 2. Overview of Breast Cancer

### 2.1. Epidemiology

BC is one of the most severe worldwide health concerns among women. A report from the Global Cancer Statistics indicated that BC is the most prevalent cancer in 185 countries [16]. BC in women has the highest number of new cases, making up 11.6% of all cancer diagnoses, followed by lung cancer at 11.4% and colorectal cancer at 10.0% [16]. At the national level, according to the American Cancer Society, BC is the most prevalent cancer among women in the United States. The report predicted that there would be 297,790 cases of invasive BC and that approximately 43,700 women may die from BC in 2024 [17]. Also, BC was the leading cause of cancer death among black women in 2019 [18]. The incidence of BC has been steadily increasing by 0.5% annually from 2010 to 2019 [17,19]. Nevertheless, due to increased health awareness, advanced treatment, and evolving research, the mortality rate of BC has declined since 1989 [17]. 

### 2.2. BC Types and Heterogeneity

BC is a complex and heterogeneous disease that has different clinical presentations in individuals and varied responses to treatment due to the fact that cancer can arise in any random cell of the mammary gland and due to its diverse pathological characteristics [20]. Breast carcinoma, often referred to as adenocarcinoma, can manifest either non-invasively or invasively, affecting different locations of breast tissues. Based on its pathological characteristics, BC can be categorized into basal-like and non-basal-like BC. Non-basal-like BC can be further classified into subtypes such as luminal A, luminal B, and HER2-enriched based on the expression levels of estrogen receptor (ER), progesterone receptor (PR), and human epidermal growth factor receptor 2 (HER2) [21]. One special case of basal-like BC is triple-negative BC (TNBC), identified by the negative expression of ER, PR, and HER2 [22]. This distinct molecular feature makes TNBC more aggressive and lacks effective treatment strategies. TNBC stands out for its high proliferation rate, abnormal changes in DNA repair genes, and heightened genomic instability. 

Breast carcinoma can also be classified into invasive ductal carcinoma (IDC) and ductal carcinoma in situ (DCIS). IDC is the most prevalent type that accounts for almost 55% of diagnosed BC and can invade the surrounding tissues, while DCIS is considered a precancerous lesion that has the potential to progress if left untreated [20]. IDC can be further stratified based on the tumor size, nuclear grade, lymph node invasion, and levels of ER and PR expression [23]. Due to the heterogeneity of IDC, many factors such as biological features lead to different sensitivity and responses to BC treatment. There are abundant lymphatic vessels around the nipples, making the cancer cells prone to experiencing metastasis through lymphatic vessels [24]. Invasive lobular carcinoma (ILC) is less common than IDC and occurs in approximately 8–15% of all diagnosed BC. The morphology of lobular carcinoma is different from ductal carcinoma. ILC is characterized by small cells with loose cell–cell adhesion caused by the lack of E-cadherin expression, which is a distinguishing feature of ILC for diagnosis [25]. Thus, screening ILC can be difficult and inaccurate due to its distinct morphology and absence of findings on mammography. ILC tumors tend to exhibit a large size, more significant differentiation, and positivity of ER [26]. ILC often metastasizes to the bones, gastrointestinal tract, and ovaries. while metastasis in lung and lymph nodes is less common [26,27,28]. 

### 2.3. Risk Factors of BC

Many modifiable and non-modifiable risk factors are associated with BC. The risk of developing BC tends to increase with aging. Studies have shown that almost 99.3% and 71.2% of BC cases occur among females over 40 and 60, respectively [29]. Invasive BC and DCIS demonstrate an exponential increase until the age of 50, followed by a slight increase afterward [30]. Also, there are regional and ethnic variations in age distribution. In Latin America, a notable proportion of BC cases arise between ages 20 and 44 [31]. Racially, African Americans (AAs) have higher BC mortality than Caucasian women. Despite social disparities, AAs are more likely to develop advanced-stage BC [32].

Family history is associated with BC risk. Females whose first-degree relatives have BC are likely to develop BC in their lifetimes [33]. Inherited BC is usually associated with genetic mutations in genes such as *BRCA1/2*. *BRCA1* and *BRCA2* are a pair of tumor-suppressor genes responsible for regulating cell growth and preventing uncontrolled cell division. The *BRCA1* gene plays an important role in activating the checkpoint and DNA repair by binding to the DNA complex, whereas the *BRCA2* gene is a crucial mediator for homologous recombination, a fundamental mechanism in DNA repair [34]. Either germline or somatic mutations in these genes are related to an increased risk of developing BC [35,36]. 

As age increases, environmental factors replay major roles during BC carcinogenesis [37]. In addition, prolonged estrogen exposure is one of the major contributing factors to BC. For example, females who have early menarche (<12 years), menopause after 50 years of age, first childbirth over 30 years of age, or long-term menopausal estrogen therapy have an elevated risk of developing BC compared to their relative counterparts [9,38]. 

Lifestyle, including fitness and nutrition, is a significant modifiable factor and is vital in affecting the risk of BC. It has been well documented that excessive alcohol intake and dietary fat intake raise the risks of BC. Alcohol can activate the ER pathway by increasing the circulating estrogen [39]. A study revealed that drinking around 35 g of alcohol per day is associated with a 32% increased risk of developing BC [40]. The increased consumption of saturated fat and trans fat is also associated with a higher BC mortality rate [41]. Being overweight or obese is closely related to an increased risk of postmenopausal BC, possibly due to the higher levels of circulating estrogen secreted from adipose tissue with age [42]. Modifiable risk factors for BC encompass a range of socioeconomic and lifestyle factors. Socioeconomic statuses, characterized by income, education level, and insurance status, also significantly influence BC survival rates [43,44].

## 3. BC Treatment

BC treatment has undergone significant evolution over the past decades. The treatment strategies have transformed from the standardized approach to personalized medicine. Advanced diagnostic tools such as molecular imaging and genomic expression profiles facilitate more precise tumor characterization and promote multidisciplinary management to mitigate treatment-induced side effects and the risk of recurrence [45,46].

BC treatment encompasses a range of modalities, including surgery, chemotherapy, radiotherapy, endocrine therapy, targeted therapy, and immunotherapy [47]. Surgery is considered the primary treatment option for non-metastatic BC, aimed at removing the tumor and potentially affected lymph nodes. Chemotherapy-based systemic therapy is used in the case of metastatic BC to reduce tumor size, enabling breast-conserving surgery and reducing the necessity for axillary lymph node dissection [47]. Systemic treatment reduces the locoregional recurrence rate in ER+/HER+ BC and TNBC. Chemotherapy, including neoadjuvant chemotherapy (NAC), adjuvant chemotherapy (AC), and salvage chemotherapy, also plays an important role in managing metastatic BC. Both NAC and AC are applied for patients with large tumor sizes and low expression of HER [47,48]. Radiotherapy, involving the use of high-energy rays or particles, is a common post-surgery treatment for BC.

In addition to conventional cancer treatment approaches, several other treatment options are available for BC that are tailored to the specific molecular characteristics of the cancer cells, including endocrine therapy, targeted therapy, and immunotherapy. Endocrine therapy plays a pivotal role in the management of hormone receptor-positive (HR+) BC, where cancer cells possess receptors for estrogen or progesterone [48]. It aims to disrupt the hormonal signaling pathways that drive the growth and proliferation of HR+ BC cells. Approaches include ovarian suppression or ablation in premenopausal women, aromatase inhibitors (AIs) in postmenopausal women, selective estrogen receptor modulators like tamoxifen to oppose ER function, and fulvestrant to reduce ER expression. Tamoxifen has been extensively studied for its effectiveness in treating BC at an early stage by lowering the recurrence rate and increasing the survival time [49,50]. In addition, the inhibition of aromatase, which is used to produce estrogen in the ovaries, is another potential therapeutic strategy in both adjuvant and first-line metastatic conditions. Numerous studies have compared the efficacy of tamoxifen and AIs in postmenopausal patients and shown that AI treatment reduced the recurrence rate more effectively than tamoxifen treatment [51,52,53]. However, while AIs are effective in blocking estrogen production and suppressing tumor growth, some tumors develop resistance to these drugs over time. 

Targeted therapies are designed to interfere with specific molecules or pathways that are critical for the growth and survival of BC cells. They often focus on molecular targets such as HER2, CDK4/6, and the PI3K/AKT/mTOR pathways [48]. The most effective targeted therapy is the anti-HER2 receptor monoclonal antibody (trastuzumab) that attaches to HER2 receptors and blocks the signals that promote cancer cell growth and survival [47]. Many studies have explored the efficacy of synergistic treatment combining AIs and targeted therapies [54,55]. The combined treatment of trastuzumab and letrozole showed durable responses lasting at least one year in patients with advanced BC [54]. Compared to letrozole alone, the addition of lapatinib was well tolerated and resulted in a notably longer progression-free survival time and higher objective response rate [55].

Immune checkpoint inhibitor (ICI)-related immunotherapies targeting immune checkpoint molecules such as programmed cell death 1 (PD-1) and its ligand programmed death ligand-1 (PD-L1) have exhibited promising clinical benefits in patients who may not respond well to traditional treatments. Recently, ICI-related immunotherapies such as pembrolizumab (anti-PD-1 antibody) and atezolizumab (anti-PD-L1 antibody) have been approved as a first-line treatment along with chemotherapy for early-stage and advanced TNBC patients [56,57].

Despite recent advances in BC treatments, exploration of a convenient, nontoxic, and efficacious approach is among the hottest areas in cancer research. Accumulating evidence has shown that bioactive dietary compounds can serve as adjuvant therapies in addition to conventional treatments for BC treatment by acting as anticancer agents through the inhibition of cell proliferation, the induction of cell cycle arrest, and apoptosis in tumor cells [14]. For example, polyphenols in fruits and vegetables have shown antioxidant, anti-inflammatory, and antimicrobial properties against BC. It is believed that the adoption of healthy dietary patterns containing high amounts of bioactive compounds can enhance the efficacy of conventional treatments, leading to improved treatment outcomes and reduced recurrence and mortality rates that provide long-term benefits for BC patients and survivors. 

## 4. BC and Microbiome

### 4.1. Gut Microbiome

The human microbiome contributes to many physiological processes. Over 100 trillion microbes are located in different sites like the gut, skin, respiratory tract, etc. [58]. The gut microbiome expresses approximately 3.3 million genes, indicating the essential roles of the microbiome in modulating biological functions [59]. The gut microbes are involved in food digestion, nutrient absorption, immune system maintenance, and homeostasis [60]. Maintaining the symbiotic relationship between the host and microbiome is important to ensure gut homeostasis and overall health [61]. Dysbiosis, characterized by the imbalanced composition and types of the microbial community, may increase susceptibility to various chronic human diseases, including obesity, diabetes, and cancer, via an inflammatory-mediated mechanism [62]. An imbalanced gut microbiome community likely induces inflammatory mediators that may promote tumor progression [63,64]. Alterations in the microbiome trigger the generation of toxins, inflammatory responses, and carcinogenic metabolites [62,65]. 

Dysbiosis is considered a significant contributor to the initiation and development of breast carcinoma. The disrupted gut bacteria community affects the production of anticancer metabolites and normal estrogen metabolism in the gut [66]. It is also related to an impaired immune defense system by decreasing lymphocytes and increasing neutrophils, contributing to decreased survival rates in BC patients [67]. One study found that *Methylobacterium radiotolerans* is enriched in tumor tissue, while *Sphingomonas yanoikuyae* is enriched in normal tissue. This inverse relationship suggests the role of dysbiosis in BC development. The bacterial DNA load is also reduced in tumor tissues and inversely correlated with disease severity [68].

The gut microbiome influences BC differently, playing pivotal roles in tumor development, progression, and treatment responses. Through intricate interactions with the immune system, gut bacteria can promote anti-tumor activities or foster chronic inflammation. Specific microbial taxa such as *Blautia*, *Faecalibacterium prausnitzii*, and *Bifidobacterium* are associated with different clinical stages of BC, suggesting a potential link between gut microbiota composition and disease severity [69]. Furthermore, microorganisms can metabolize dietary components such as fibers, producing bioactive compounds like short-chain fatty acids (SCFAs) that possess anti-inflammatory and anti-tumor properties. Specifically, sodium butyrate, as an important SCFA, can be produced by the phylum *Firmicutes* like *Faecalibacterium*, *Roseburia*, and *Eubacterium* [70]. Butyrate works as a histone deacetylase (HDAC) inhibitor and induces cell cycle arrest and apoptosis in BC cell lines. Also, the gut microbiota is essential for regulating steroid hormone metabolism and estrogen production, potentially influencing hormone receptor-positive BC [71]. Additionally, gut bacteria play a crucial role in regulating gut barrier function and modulating the efficacy of chemotherapy and immunotherapy in BC treatment. Collectively, these effects influence the pathogenesis of tumor cells and their microenvironment in diverse ways. The gut microbiota compositions in BC patients may vary based on the different stages and outcomes of BC. Therefore, it is crucial to understand the dynamics of this microbiome–host interaction. 

Table 1 presents the influence of selective gut microbiota on BC, highlighting both promoting and suppressing roles across different bacterial species. This table integrates findings from both in vitro and animal model studies to explore the role of the gut microbiota in BC. It examines how various bacterial species influence tumor progression, immune responses, and the tumor microenvironment. By focusing on in vitro experiments and animal models, this review elucidates the impact of specific gut bacteria on BC outcomes and potential therapeutic strategies. Specifically, *Lactobacillus* supports epithelial barrier function and modulates inflammation and tumor growth [72,73]. Its effects on promoting immune responses by regulating cytokines are also supported by animal studies [74,75,76]. *Bifidobacterium* also has anticancer effects via regulating immune activity, inhibiting angiogenesis, and inducing apoptosis, as supported by in vivo and in vitro studies [77,78,79,80]. Other bacteria like *Faecalibacterium* [81], *Akkermansia* [82,83,84], *Enterococcus* [85,86], *Clostridium* [87,88,89,90,91], and *Fusobacterium* have shown tumor-suppressing properties [92,93,94,95,96]. Conversely, *Helicobacter* and *Escherichia* are likely to promote the growth and migration of tumors [97,98,99,100]. Moreover, Bacteroides, particularly *Bacteroides fragilis* (*B. fragilis*), promote BC by inducing systemic inflammation and altering the tumor microenvironment to facilitate metastasis [101,102,103]. However, the exact mechanisms regarding how these microbes influence breast tumorigenesis remain incompletely understood.

A body of human studies provides solid evidence linking BC and gut dysbiosis. BC patients exhibit a distinct gut microbiota composition compared to those with benign tumors or healthy individuals. A study of postmenopausal BC patients showed that their gut microbial community had a significantly lower alpha diversity. The microbiota composition was significantly altered in BC compared to the healthy controls [104]. Specifically, BC patients showed more abundant bacteria species such as *Clostridiaceae, Faecalibacterium,* and *Ruminococcaceae* but lower levels of *Dorea* and *Lachnospiraceae.* Reduced diversity in the gut microbiome is linked to medical conditions such as obesity, insulin resistance, and high-level *C*-reactive protein [105]. Notably, some of these factors have established associations with increased BC risk. These studies indicate that BC patients had a less diverse gut microbiome community with different bacterial compositions, suggesting that the gut microbiome might affect BC development. Fecal microbial status may also affect the clinical stages and progression of BC. In a comparative case–control study, BC patients showed reduced microbial diversity with a higher abundance of *Firmicutes* and a reduction in *Bacteroidetes* [106]. Moreover, bacterial counts and compositions such as *Firmicutes*, *Faecalibacterium prausnitzii*, and *Blautia* varied significantly based on BMI, clinical stages, and histoprognostic grades in BC patients [69]. BC patients had more abundant bacteria species such as *Porphyromonas* and *Peptoniphilus,* and patients with benign lesions had more *Escherichia* and *Lactobacillus* [107]. A study found that dysbiosis only happened among postmenopausal patients, indicating that dysbiosis may be associated with the menopause stage [108]. 

Mechanistically, the intricate interplay between the gut microbiota and BC involves various factors, ranging from potentially detrimental pro-carcinogenic toxins to metabolites hindering BC progression. Some gut bacteria have been identified as producers of pro-carcinogenic toxins and inhabitants of breast tissue [58,101]. A study demonstrated the presence of *B. fragilis* in the colon and its ability to promote breast tumorigenesis and metastasis by activating the Notch and β-catenin signaling pathways [101]. Specifically, the *B. fragilis* toxin (BFT) released from entero-toxigenic *B. fragilis* (ETBF) was found to mediate cell migration and invasion [101]. *Fusobacterium nucleatum*, a known colon cancer activator, can translocate from the GI tract to the breast through the blood, promoting BC metastasis [95]. Its presence in BC tissues also correlates with an enhanced level of galactose-N-acetyl-D-galactosamine (Gal-GalNAc), which plays a crucial role as a molecular mediator in facilitating the binding of *F. nucleatum* to host tissues, enabling its translocation from the GI tract to the breast. In mouse models, *F. nucleatum* expressing Fap2 promotes tumor growth and metastasis, while those lacking Fap2 exhibit reduced colonization of mammary tumors. Furthermore, *F. nucleatum* inoculation suppresses the accumulation of tumor-infiltrating T cells, exacerbating tumor progression [95]. Other bacterial strains, such as *Peptostreptococcus* strains, contribute to tumor progression by inducing reactive oxygen species (ROS) synthesis [109]. 

In the context of BC tumorigenesis, the microbiome plays a key role in regulating steroid hormones like estrogen, which is the main contributor to BC progression, especially ER-positive BC [71]. After conjugation in the liver, estrogen is released through bile acid, which further enters the intestine [110]. This crucial link between estrogen metabolism and the microbiome introduces the concept of estrobolome, which represents the group of microbiome reactions related to the modulation of estrogen [111]. β-glucuronidase is a key player in estrobolome. It is responsible for deconjugating estrogen and enabling it to be bioactive and reabsorbed into the circulation. Free estrogens are predominantly produced from this deconjugation process by β-glucuronidase, produced by microbial communities from families like *Clostridia* and *Ruminococcaceae* or the genus *Escherichia* [7,91]. β-glucuronidase-producing bacteria are frequently observed to be overactive in gut dysbiosis. This heightened activity is often linked to various factors, including dietary choices, alcohol consumption, and antibiotic usage [7]. The recirculated estrogen may interact with breast tissues, resulting in cell proliferation, the onset of BC, and a higher risk of ER-positive cancers [71,112,113]. Consistently, gut bacteria with β-glucuronidase activity are abundant in BC patients, including *S. pyogenes*, *Clostridia* spp., *Bacillus* spp., and *E. coli* [67]. 

The dysregulated immune reactions to gut bacteria could potentially facilitate cancer onset in epithelial tissues far from the gastrointestinal tract [114]. A study that focused on intestinal cancers found that infection with the intestinal pathogen *Helicobacter hepaticus* up-regulated the TNF-α pro-inflammatory cytokine and increased the risk of mammary neoplasia in a mouse model, indicating that gut bacteria are associated with the systemic immune response [97]. Moreover, neutrophil depletion inhibits tumor formation and development. Those *H. hepaticus*-infected mice had fewer mammary lesions after the depletion of neutrophils, contrasting with the non-depleted control group [115]. This underscores the significance of neutrophils as crucial mediators in the distant influence of gut microbiota on mammary epithelial carcinogenesis.

Taken together, understanding the dynamics of this intricate microbiome–host interaction is crucial for unraveling the complexities of BC progression. Further research into the functional significance of these microbiota alterations in BC patients is crucial for investigating their implications for BC prognosis and treatment outcomes.

**Table 1:** Summary of bacterial species and mechanisms in breast cancer.

Bacterial Species	Research Evidence	Mechanistic Actions in BC
*Lactobacillus *	In vivo studies: Oral administration of *L. acidophilus* to mice with breast tumors significantly increases the survival time and improves the immune response by increasing the production of IFN-γ and decreasing IL-4 [74] Consumption of *L. acidophilus* decreases the breast tumor growth rate and enhances the production of IL-2 among mice with breast tumors [75] Selenium nanoparticles enriched with *L.plantarum* increase the production of IFN-γ, TNF-α, and IL-2, decrease tumor volume, and increase survival time [76]	Protect the epithelial barrier from inflammation and maintain homeostasis [73] Change the production of cytotoxin [73] Produce natural killer cells to control tumors [73] Induce epigenetic down-regulation of cancer–testis antigen expression [72] Reduce tumor growth rate [72] Increase lymphocyte proliferation [72]
*Bifidobacterium *	In vivo studies: The recombinant *Bifidobacterium* secrets a molecule that significantly suppresses tumor growth in mice with BC [78] In the BC model of mice, *B. bifidum* reduces tumor volume and increases the production of the Th1 cytokine [79] *Bifidobacterium*-derived membrane vesicles induce apoptosis of TNBC cells in a mouse model [77] *B. infantis* milk in combination with doxorubicin decrease the breast tumor volume in a murine model [80]	Exert anticancer effects, induce apoptosis, and suppress tumor growth [77] Enhance the immune function by stimulating the activation of macrophages, natural killer (NK) cells, and B lymphocytes [77] Inhibit angiogenesis and TNF-α transcription [77]
*Bacteroides *	In vivo studies: The enteric abundance of *B. fragilis* induces systemic inflammation and alters the tumor microenvironment, which promotes metastasis in the lungs and liver; it also promotes tumor development by changing the morphology and functions of BC cells [103] Human studies: In a study comparing the fecal microbiomes of BC and non-malignant BC patients, *Bacteroides* had a strong positive association with the odds of developing BC [102]	Promote tumor growth and metastasis [101] Induce epithelial hyperplasia [101] Activate β-catenin and NOTCH1 to renew cancer cells [101] Activate the human enzyme spermine oxidase and generate ROS [101]
*Faecalibacterium *	In vitro studies: *Faecalibacterium prausnitzii* inhibits the invasion and proliferation of BC cells by suppressing the IL-6/STAT3 pathway [81]	Prevent IL-6 and the phosphorylation of JAK2/STAT3 in BC cells [81] Suppress the invasion and promote the apoptosis of BC cells [81]
*Akkermansia *	Human studies: *Akkermansia* is associated with small breast tumor size and protects from diabetes and obesity [82] In vivo studies: *Akkermansia* induces anticancer activities and enhances immune responses to lung and kidney cancer [83]	Prevent metabolic syndrome and obesity [84] Enhance the concentration of anti-inflammatory factors [84] Promote anticancer activities [84]
*Enterococcus*	In vitro studies: *E. faecalis* induces apoptosis and suppresses tumor growth in BC by up-regulating the tumor suppressor protein [85] *E. faecalis* exerts cytotoxicity effects on BC cells, reduces cancer cell viability, and significantly decreases cell proliferation [86]	Produce extracellular oxygen-derived species and hydrogen sulfide and cause DNA mutations [85] Suppress proliferation and induce apoptosis [85]
*Clostridium *	In vitro study: The recombinant *Clostridium difficile* toxin B (rcdtB) inhibites tumor growth, induces apoptosis, decreases cell migration, and activates inflammatory responses [87] *Clostridium histolyticum* reduces BC cell proliferation and limits cell migration [88] In vivo studies: *Clostridium novyi* has the potential to completely cure tumors smaller than 1000 mm [3] in a mice BC model [89]	Induce cell death through both necrosis and apoptosis [90] Induce long-term anti-tumor immunity [90] Produce lithocholic acid to inhibit the proliferation of BC cells [91]
*Fusobacterium *	In vitro studies: The *F. nucleatum* gDNA levels in BC tissues are significantly elevated and positively related to tumor growth and migration, improving cell viability by regulating TLR4 [92] *F. nucleatum* colonizes BC tissues with elevated Gal-GalNAc levels [93]	Produce extracellular oxygen-derived species and hydrogen sulfide and cause DNA mutations [94] Influence the infiltration of tumor-infiltrating lymphocytes and inhibit NK cells [95] Change the tumor immune microenvironment [96] Suppress T-cell infiltration and promote mammary tumor growth [96]
*Helicobacter *	In vivo studies: Mice infected with *Helicobacter hepaticus* exhibit increased mammary carcinoma and enhanced intestinal adenoma multiplicity by up-regulating TNF-α [97]	Up-regulate TNF-α [97] Accelerate breast tumor growth [97] Activate the human enzyme spermine oxidase and generate ROS [97]
*Escherichia*	In vitro studies: *E. coli* has a high relative abundance in BC microenvironment and causes DNA double-stranded breaks [99] *E. coli* has oncogenic properties and supports BC cell pathologies; *E. coli* secretome controls energy metabolism by increasing de novo pyrimidine, fructose, and mannose synthesis [100]	Control energy metabolism of BC cells and change the tumor microenvironment [100] Specific secretomes are oncogenic [100]

Table 1 presents a concise overview of the bacterial species implicated in BC and their corresponding mechanisms of action. Each row of the table corresponds to a specific bacterial species, with the columns detailing the mechanisms through which these bacteria contribute to BC progression.

### 4.2. Breast Microbiome

Although most of the human microbiome is located in the GI system, recent studies have suggested that the female mammary glands also harbor a special group of bacterial species. Skin or oral bacteria may serve as a potential origin of breast tissue microbiota as they can migrate through nipple–areolar orifices, gaining access to the breast tissue. This contrasts with the previous idea that the breast tissues are sterile. Understanding the microbiome in breast tissues helps explore the carcinogenic mechanisms of BC. 

The microbial composition of BC tissues is closely linked to tumor progression. Although it is an evolving area, many studies indicate that the human breast microbiome significantly varies between BC patients and healthy individuals [116]. The microenvironment in normal breast tissues includes the epithelial, interstitial, and mucosal immune systems. Consequently, inflammation can arise due to alterations in this microbial milieu caused by bacterial infections. Thus, the presence of immune responses within the breast microenvironment indicates the involvement of the breast microbial community [117]. Breast tissues embrace a relatively diverse microbiota [118]. The viability of breast microbiota suggests that dysbiosis may significantly contribute to the development of BC and metastasis [101]. Distinct from other body sites, the major species of the breast microbiota are *Proteobacteria* and *Firmicutes*, which may be attributed to the host microbiome’s adjustment to the nutrient-rich fatty composition of female breast [68,119]. Other microbiota that exist in mammary tissues include *Bacillus sp*., *Enterobacteriaceae* sp., and *Staphylococcus* sp. [119].

One study found that tumor tissues had a unique microbiota composition, with more *Methylobacterium radiotolerans* but less *Sphingomonas yanoikuyae* than the paired normal tissue [68]. In the same study, the results showed an inverse association between the bacteria load and severity of BC, potentially holding diagnostic significance. Such dysbiosis may create an environment for breast tumorigenesis due to the reduced bacteria load and the increased abundance of bacteria like *S. yanoikuyae* [68]. Additionally, another study showed that BC patients had more abundant *Bacillus, Enterobacteriaceae*, and *Staphylococcus* but less *Lactobacillus* in their tumors compared to paired normal tissue around breast tumors from the same patient [119]. Differences also exist between BC-adjacent normal tissue and normal mammary tissue. For example, *Lactobacillus*, *Acetobacterraceae*, and *Xanthomonadaceae* are less abundant in BC-adjacent normal tissue than normal mammary glands [120]. Nonetheless, no significant distinctions were observed between the adjacent and tumor tissues, suggesting a similar bacterial distribution around the tumor area. Moreover, tumor tissues exhibited significantly lower α-diversity compared to the adjacent and healthy tissues [116]. Interestingly, the reduced bacteria in tumor tissues aligned with the diminished expression of approximately one-third of the studied antibacterial response genes [68]. 

BC subtypes, such as luminal A, luminal B, HER2+, ER+, and TNBC, exhibit distinct microbiota compositions [116]. One study indicated that the breast tissues of BC patients contain more bacteria such as *E. coli* and *Staphylococcus epidermidis* that cause DNA breaks in vitro compared to healthy individuals [99]. In the same study, *Bacillus* was found in higher concentrations in BC patients and had carcinogenic properties [99]. The species *Bacillus cereus* plays a role in metabolizing the hormone progesterone into 5-alpha-pregnane-3,20-dione (5αP), which usually presents in cancerous breast tissues and promotes cell proliferation [121]. Moreover, *B. fragilis* is identified in cancerous breast tissues and shows pro-oncogenic effects on BC progression. Together with ETBF, *B. fragilis* triggers mammary gland hyperplasia and systemic inflammation, along with the morphological changes leading to the adoption of phenotype with enhanced migratory and invasive properties [101]. 

## 5. Nutrition Intervention in BC

### 5.1. Dietary Patterns

Nutrition intervention plays a crucial role in the comprehensive management of BC, encompassing dietary strategies and incorporating bioactive compounds to support overall health and potentially improve treatment outcomes. Among the modifiable factors, the dietary pattern is especially important to the risk of BC, the post-diagnosis outcomes, and the survival rate. An increasing body of evidence suggests that adhering to a healthy dietary pattern with a high intake of fruit, vegetables, whole grains, poultry, and fish, along with appropriate weight management strategies and physical activity may be protective against the onset of BC [9,122]. On the contrary, excessive consumption of a Western diet featuring refined starches, alcohol, red meat, and sweetened and high-fat, highly processed food is a major risk factor for BC, primarily due to adipose tissue inflammation, creating an environment suitable for BC development [122,123,124]. Additionally, another study compared the association between the Western diet and Mediterranean diet, and mammographic density, a biomarker representing a higher BC risk at elevated levels, showed that participants who followed the Western diet were more likely to have high mammographic density, especially in obese or overweight individuals [125]. In Table 2, epidemiological studies are included to demonstrate the association between different dietary patterns and BC risk and mortality. These studies highlight the importance of adopting healthy dietary patterns for the prevention of BC.

#### 5.1.1. Mediterranean Diet (MD)

The MD is considered to be one of the healthy dietary patterns that effectively support cancer prevention and treatment strategies. It emphasizes the intake of plant-based food, whole grains, and healthy fats, and limits red meat and highly processed food intake. It has been shown to have protective effects on reducing BC risk and mortality, particularly for TNBC. A possible reason for this is the potential impacts of beneficial nutrients such as polyphenols, healthy fatty acids, and micronutrients on the inhibition of inflammation, DNA damage, and oxidative stress [126,127,128]. Table 2 summarizes studies on the association between BC and adherence to the MD. Low adherence to the MD is linked to a 13% higher risk of all-cause mortality compared to medium adherence [124]. Higher adherence to the MD is associated with a 15-year overall survival rate of 63.1% compared to 53.6% for low adherence [126]. It also correlates with improved physical functioning, well-being, and lower symptomatic pain scores [129]. Some studies showed an inverse association between healthy fatty acids, such as monounsaturated fatty acids (MUFAs) in olive oil, docosahexaenoic acid (DHA), eicosapentaenoic acid (EPA), and other long-chain polyunsaturated fatty acids (PUFAs), and BC risk and prognosis due to reduced inflammation and oxidative stress [126,130,131]. 

Moreover, the adoption of the MD is associated with increased diversity in the gut microbiome and a distinct composition of gut microbes compared to the Western diet. This may be attributed to the high fiber content in the MD, which promotes the beneficial microbial populations in the gut [132]. The MD is associated with a reduction in dysbiosis and bacteria such as *E. coli* while favoring a higher percentage of *Bifidobacteria* and SCFA-producing bacteria. The MD may also affect the mammary gland-specific microbiota and metabolites. One animal study showed that following the MD led to a higher abundance of beneficial microbes such as *Lactobacillus* and bile acid metabolites and decreased *Ruminococcus* and *Coprococcus* in mammary glands and feces compared to those following the Western diet [133]. This indicates that adhering to the MD benefits biodiversity and microbial activities, implying potential anticancer effects attributed to the diet on the microbiome specific to breast tissue. Additionally, in the same study, the MD group demonstrated a higher level of phenolic compounds in the mammary glands, including the metabolites of tyrosine, tryptophan, and phenylalanine that may support the effects of bacteria on generating metabolites [133]. Similar for its health benefits, the Atlantic Diet, common along the coasts of Spain and Portugal, emphasizes fish, particularly rich in omega-3 fatty acids, alongside local fruits, vegetables, and moderate wine consumption. This diet also underscores the potential of dietary patterns in mitigating cancer risks, particularly through nutrients that reduce inflammation and oxidative stress, which are critical factors in cancer progression [134].

#### 5.1.2. Ketogenic Diet (KD)

Additionally, the impact of the KD on BC risk and progression has become a subject of interest in contemporary research. The KD is characterized by low carbohydrate and high fat intake, along with a moderate level of protein intake [135]. This dietary pattern leads to the production of ketone bodies and better control of the blood glucose levels [136]. According to the Warburg effect, unlike normal cells, cancer cells tend to rely on glycolysis and increase the uptake of glucose [137]. Ketone bodies, specifically β-hydroxybutyrate (β-HB), possess anti-inflammatory properties that are vital in tumor development [138]. Moreover, ROS can be regulated and balanced by the glutathione and the transcription factor, nuclear factor erythroid 2-related factor 2 (Nrf2), which is activated by the KD, supporting the protective effects of the KD [139]. 

A number of studies show that the KD is a potent choice for cancer treatment because of its effects on delaying tumor initiation and growth [140]. In an animal model, following the KD reduced serum insulin and inhibited breast tumor growth and lung metastasis growth [137,141]. The KD also demonstrates the potential to enhance the treatment efficacy of anticancer therapeutic drugs and improve quality of life and metabolic health [138,141,142,143,144]. One animal study showed the effects of the KD combined with probiotics on reducing tumor size. The combination therapy inhibited tumorigenesis by increasing the blood levels of β-HB and reducing serum IGF-1 [145]. Clinically, patients undergoing chemotherapy along with KD consumption can safely achieve nutritional ketosis, leading to improvements in body composition and insulin resistance [146,147]. Also, patients following the KD had reduced tumor size compared to the control group. A notable reduction in the disease stage was observed in patients with locally advanced conditions within the KD group [145,148,149]. More studies, especially randomized controlled studies and clinical trials, are necessary to further investigate the role of the KD as a preventative measure and treatment strategy for women with different types of BC. 

While the main effect of the KD is on serum glucose and BMI, the KD is also associated with changes in microbial profiles in the gut. The low content of nondigestible carbohydrates leads to reduced bacterial abundance. In addition, because of the reduced blood glucose and increased ketone bodies, the KD reduced abundances of pro-inflammatory bacteria like *Desulfovibrio* and *Proteobacteria* [150,151] as well as the abundance of *Bifidobacterium*, leading to a reduction in intestinal pro-inflammatory Th17 cells [152]. Another significant observation is an overall reduction in gut microbial diversity. Moreover, specific bacterial strains such as *Roseburia*, *Eubacterium rectale*, and *Bifidobacterium* tend to decrease with adherence to the KD. Alongside the decline in beneficial bacteria, there is also a reduction in SCFA levels [153]. 

#### 5.1.3. Plant-Based Diet (PD)

A PD encompasses a wide range of eating patterns characterized by the limited consumption of animal products and a regular intake of plant-based foods. The relationship between BC and PDs, including vegan and vegetarian diets, is a topic of ongoing research. Several studies showed an association between low meat intake or a PD and decreased total cancer and BC mortality [154,155,156,157,158,159,160]. However, the results have been inconsistent [161,162]. Fruits and vegetables contain high levels of fiber and many other antioxidant, anti-carcinogenic, and bioactive phytochemicals, including carotenoids, flavonoids, terpenes, phytoestrogen, polyphenols, and phenolic acids [123,163,164,165]. Studies have shown that polyphenols exhibit potent anti-inflammatory properties against a variety of human chronic diseases [166]. Specifically, polyphenols have been studied for their roles as AIs to interact with estrogen-signaling pathways by reducing the production of estrogen [167]. They also regulate the autophagy processes and important signal pathways like PI3K/AKT, RAS/RAF/ERK, and AMPK [168]. PDs may be beneficial for BC patients due to their effects on influencing important tumor-related genes and/or signal pathways, including cyclooxygenase-2 (COX-2), transcription factor NF-κB, and ER signaling [166,169]. Both the overexpression of COX-2 and the activation of the NF-κB and ER pathways are associated with promoted tumor growth, facilitating the high-grade and invasive type of BC [170,171]. 

PDs have major effects on the gut microbiome. Polyphenols increase the level of beneficial bacteria like *Bifidobacterium* and *Lactobacillus*, and microbial metabolites such as SCFAs. Pectin, commonly found in plant cell walls, promotes the growth of butyrate-producing bacteria like *Clostridium cluster XIV* [172]. Inulin, a starchy substance found in a wide variety of fruits and vegetables, acts as a prebiotic and increases *Bifidobacterium* and *Faecalibacterium prausnitzii* [173]. Both pectin and inulin demonstrate anti-inflammatory properties and increase overall bacterial diversity. PDs also decrease the ratio of *Firmicutes* to *Bacteroidetes* [174]. Moreover, one study found that fruit and vegetable supplementation led to a reduction in the relative abundance of the *Erysipelotrichaceae* and *Lachnospiraceae* families, including *Ruminococcus* and *Lachnobacterium*, that are correlated with inhibitory effects on pro-inflammatory responses [175]. Other studies investigating the effects of PDs on gut microbiome composition are presented in Table 2 [176,177,178].

The excessive consumption of red meat remains a risk factor for BC due to its rich amount of heme iron, saturated fat, and carcinogens that may be increased during cooking practices [179,180]. Under high temperatures, carcinogens like heterocyclic amines, *N*-nitroso compounds, and polycyclic aromatic hydrocarbons may be produced, causing inflammation and oxidative stress that may facilitate BC development [181,182]. Moreover, heterocyclic amines have been shown to possess estrogenic properties and influence hormonal signaling pathways involved in cell proliferation and differentiation [183]. This association between heterocyclic amines and BC is also validated in human cohort and animal studies [184,185]. High meat intake also promotes the growth of *Roseburia*, *Faecalibacterium*, and *Blautia* from the family *Lachnospiraceae* [186]. Another study indicated that high protein intake reduced the proportion of the *Roseburia* and *Eubacterium rectale* group, known for its butyrate-producing capabilities, along with the reduced phenolic acids that have antioxidant activities [187]. High animal protein intake may be related to an increased abundance of pathogenic bacteria, while plant protein increases beneficial bacteria [188,189,190,191]. The increased abundance of pathogens and content like heme iron associated with high meat consumption may contribute to chronic inflammation and DNA damage, which are hallmarks of cancer development [192]. Moreover, gut homeostasis may be disrupted and increase susceptibility to carcinogenesis. Several studies explored the association between animal and plant protein intake and BC [193,194,195]. Therefore, prioritizing PDs while minimizing the intake of red meat could be a proactive step towards lowering the likelihood of BC occurrence.

#### 5.1.4. Dietary Approaches to Stop Hypertension (DASH) Diet 

The DASH diet promotes a high intake of fruits, vegetables, whole grains, nuts, legumes, and moderate low-fat dairy consumption. It advises limiting sodium, sugar-sweetened beverages, and red meats to manage hypertension and support heart health. Several studies revealed an inverse association between DASH diet adherence and BC risk [196,197,198,199,200]. One significant aspect of the DASH diet is its low sodium intake. An in vitro study showed that high salt intake accelerates BC progression and promotes lung metastasis, along with an increased level of Th17 cells [201]. Some other diets similar to the DASH diet that focused on the regular intake of fruits and vegetables and the low intake of red meat also showed protective effects on BC [8,202]. Similar to other PDs, the potential protective effect of the DASH diet against BC may be attributed to its rich concentration of bioactive compounds, including antioxidants like vitamin C, phenols, carotenoids, and flavonoids, as well as minerals and dietary fiber [200]. Many epidemiological studies reveal an inverse association between vitamin C intake and BC incidence and mortality [203,204]. A high dose of vitamin C supplementation may induce tumor cell death in vitro because of its pro-oxidant effect [205]. Moreover, high doses of vitamin C have demonstrated anti-proliferative effects on various drug-resistant BC cells and decreased cell viability [206]. Vitamin C also shifts gut bacteria populations and protects the intestinal barrier. In healthy individuals, a high dose of vitamin C reduces *Bacteroidetes*, *Enterococci*, and *Gemmiger formicilis*, but increases *Lachnospiraceae*, which is known to generate SCFAs and exert anti-inflammatory effects [207]. 

Moreover, the high fiber intake advocated by the DASH diet leads to a decreased risk of BC among premenopausal patients, particularly ER+/PR- BC [208]. Fermented fibers like pectin and inulin undergo fermentation by the gut microbiome to produce SCFAs [209,210,211]. Dietary fiber may contribute to a reduction in intestinal β-glucuronidase activity, which is essential for the hydrolysis of conjugated estrogens prior to absorption [212]. This process leads to the decreased reabsorption of estrogens, potentially influencing hormone levels and reducing BC risk. 

The DASH diet is also low in cholesterol, saturated fat, and total fat. The overconsumption of fat, especially animal fat from red meat and dairy products, is directly linked to a higher risk of obesity, which is one of the major contributors to BC [213]. Specifically, the accumulated adipose tissue contributes to the development of metabolic syndrome and tumorigenesis through pathways that may involve insulin and IGF-1 [214]. The excessive accumulation of lipids in adipose tissue triggers significant alterations characterized by adipocyte death and macrophages, leading to a higher risk of chronic low-grade inflammation prevalent in breast adipose tissue. Adipose tissues also play important roles in generating steroid hormones like estrogen and lipid storage. Excessive fat intake will lead to alterations, thereby increasing BC incidence [215].

High fat intake is usually associated with a reduction in *Bacteroidetes* and an increase in *Firmicutes*. Mice with high fat intake have an increased abundance of *Helicobacteraceae*, *Lactobacillaceae*, *Enterobacteriaceae*, and *Clostridiaceae* [216]. On the contrary, the DASH diet could potentially lead to a decrease in the levels of certain gut bacteria such as *Fusobacterium*, *Porphyromonas*, *Bacteroides*, *Bifidobacterium*, and *Succinivibrio*, and an increased presence of *Clostridium*, *Ruminococcus*, and *Lactobacillus* [217]. More research is needed to confirm the precise effects of the DASH diet on gut microbiota composition and how these changes may influence health outcomes.

**Table 2:** Dietary patterns and their connection with BC and bacterial regulation.

Dietary Patterns	Connection with Bacterial Regulation	Association with BC
Mediterranean diet	↑ *Bacteroides*, *Clostridium cluster XIVa*, *Prevotella*, *Roseburia*, *Ruminococcus*, *Lactobacilli*, *Bifidobacteria*, and *Faecalibacterium prausnitzii* [132] ↓ *Firmicutes* and *Proteobacteria* [132]	A low adherence to the MD, in contrast to medium adherence, correlated with a 13% increased risk of all-cause mortality [127] Following the MD is linked to improved outcomes, with a 15-year overall survival rate of 63.1% for individuals with high adherence compared to 53.6% for those with low adherence [218] Greater adherence to the MD is associated with better physical functioning and well-being and lower scores on the symptomatic pain scale [129]
Ketogenic diet	↓ *Desulfovibrio* and *Proteobacteria* [150] ↑ *Akkermansia muciniphila*, *Parabacteroides*, *Escherichia coli*, and *Lactobacillus* [150] ↓ Total bacterial count, *Roseburia*, *Eubacterium rectale*, and *Bifidobacterium* [153] ↓ Total SCFAs, acetate, butyrate, isovalerate, propionate, valerate [153]	In patients undergoing chemotherapy, patients in the KD group maintained ketosis and decreased weight, plasma glucose, plasma insulin, and insulin resistance [147] In the KD group, patients showed decreased serum insulin levels and tumor size reduction compared to the control group; patients experienced a significant decrease in disease stage [148] The KD group experienced significant reductions in BMI, body weight, body fat, and fasting glucose level compared to baseline levels [149]
Plant-based diets	↑ *Bacteroidetes*, *Prevotella*, *Roseburia*, *Bifidobacterium* and *Lactobacillus*, *Clostridium*, *Ruminococcus*, *Eubacterium rectale*, and *Faecalibacterium prausnitzii* [176,177,178] ↓ *Firmicutes*, *Bacteroidetes*, and *Bifidobacterium* [176,178]	Women with greater adherence to healthy PDs had a lower risk of BC [157] A prudent dietary pattern is associated with lower BC risk [158,159] A high intake of fruits and vegetables is related to decreased BC risk, especially with aggressive tumors [160]
DASH diet	↑ *Clostridium*, *Ruminococcus*, *Lactobacillus* [217] ↓ *Fusobacterium*, *Porphyromonas*, *Bacteroides*, *Bifidobacterium*, *Succinivibrio* [217]	In premenopausal and postmenopausal patients, DASH diet adherence decreased the BC risk [199] In case–control studies, a significant association between adherence to the DASH diet and BC risk is observed [196,200] Following the DASH diet was related to reduced non-breast-cancer mortality [197] The inverse association between DASH diet and odds of BC among Iranian women is found [198]

↑ Increased changes; ↓ Decreased changes

### 5.2. Bioactive Compounds

The role of nutrition in BC development and progression has drawn increasing interest in cancer research. Dietary components have been found to influence various aspects of BC pathophysiology, including cell proliferation, inflammation, oxidative stress, and hormone regulation. Numerous studies have focused on specific bioactive dietary compounds or phytochemicals in healthy foods such as fruit and vegetables as well as special functional foods and their roles in cancer prevention and therapeutic effects. Researchers also found that specific dietary bioactive components can increase conventional treatment efficacy or resensitize resistant BC [219]. The demand for the use of bioactive compounds as alternative adjuvant approaches for cancer prevention and therapy has been exponentially increased due to their outstanding chemopreventive characteristics and minimal side effects [14,220].

#### 5.2.1. Soy Isoflavone

Soybean products are known for their rich contents of bioactive isoflavones, which have a chemical structure similar to estrogen, so-called phytoestrogens [220]. Specifically, there are three types of isoflavone aglycones in soybean products, including genistein, daidzein, and glycitein [221]. They share a similar structure to endogenous estrogen, 17-β estradiol, but with less ability to bind and activate estrogen receptors [222]. Genistein is the most enriched isoflavone in soybean products and is a known anti-tumor agent for its effects on preventing angiogenesis and cell proliferation by inhibiting the RTK pathway and NF-κB activation [223,224]. 

Soy isoflavones can induce ER-mediated tumor inhibitory effects by primarily binding to ERβ in BC [225]. Many other mechanisms involved in genistein-induced tumor suppression effects include interrupting the activity of enzymes like tyrosine protein kinase and mitogen-activated kinase, influencing epigenetics and DNA polymerase II [221]. Human clinical trials indicate that higher isoflavone intake led to a reduced risk of developing BC by changing the hormone concentration and the duration of the menstrual cycle [226,227,228]. In Asian countries where consuming soy products is popular, studies found an inverse association between soy consumption and BC risk, especially among premenopausal women and those with a large BMI [229]. In a study involving 17 patients treated with soy isoflavone tablets for two weeks, researchers found that while the apoptosis ratios in isoflavone-treated cancer specimens did not differ significantly from untreated specimens, there was a trend suggesting the potential inhibition of cancer growth. However, ex vivo and in vitro assays using serum samples before and after treatment showed no significant changes in BC cell or endothelial cell proliferation [230].

The timing of consuming soy food is potentially influential. Studies suggest that early consumption of soy food during childhood and adolescence exhibited more potent protective effects on reducing BC risk compared to soy intake during adulthood due to the beneficial effects of soy diet exposure during the critical mammary development stages [231,232,233,234]. Our laboratory also found that maternal intake of a genistein-rich soy diet led to transgenerational protection against BC late in life in mouse offspring through epigenetic inheritance [235]. 

Moreover, genistein improved the response of breast tumors to the first-line anti-hormone treatment tamoxifen through a synergistic inhibitory effect on ER+/HER2 BC cells [236]. We observed that a combination treatment of genistein with the HDAC inhibitor trichostatin A (TSA) effectively enhanced the treatment efficacy of tamoxifen through reactivated ERα expression in ER-negative BC cells [237]. 

However, results on the effects of isoflavones on BC risk remain controversial due to concerns about the potential ER stimulatory effects on BC of soy phytochemicals. One in vitro study showed that the adverse effects of genistein may promote the growth of estrogen-dependent human breast cancer (MCF-7) cells [238]. However, because of the limitation of cell culture conditions wherein additional genistein is the sole estrogen source fueling cancer cells, those in vitro studies do not reflect the actual functions of genistein in vivo. In fact, genistein primarily binds to ERβ, leading to a tumor-suppressive effect in vivo because of its over 1000-fold weaker binding ability to ERα than endogenous estradiol. In addition, numerous epidemiological and lab studies strongly support the overall protection of soybean isoflavones in the prevention of BC, reducing the recurrence and mortality of all types of BC. Thus, soy food is clinically safe and beneficial to BC patients and survivors [239].

Soy product intake also benefits the gut microbial community by reversing dysbiosis and reducing the risk of chronic diseases [240]. For example, soy food intake may increase the abundance of probiotics such as *Lactobacilli* and *Bifidobacteria* and the *Firmicutes/Bacteroidetes* ratio [241]. One study investigated the gut microbiome of soy genistein-fed mice and the effects on tumor growth. A distinct clustering was found between the genistein-fed mice and the control group. The gut microbiomes of treated mice showed an increased abundance of *Lactococcus* and *Eubacterium* genera and the *Lachnospiraceae* and *Ruminococcaceae* family, along with significant differences in *Verrucomicrobia*. Additionally, the genistein-fed mice had a decreased breast tumor size and a 25% increase in latency of tumor growth [240]. Another study showed that genistein might have protective effects against the recurrence of mammary tumors among offspring, especially under exposure to a high-fat diet, by reducing inflammatory *Enterobacteriaceae* and increasing the levels of SCFAs and anti-inflammatory *Clostridiaceae* [242]. Our recent studies found that a maternal soybean diet can prevent high-fat-induced BC in mice offspring by significantly increasing the beneficial bacteria genus *Bifidobacterium* and beneficial metabolites such as sodium butyrate [235], suggesting that maternal genistein consumption may facilitate a favorable environment within the gut microbiota, contributing to BC prevention in offspring later in life. The potential associations between soy isoflavone intake and shifts in microbial composition are summarized in Table 3 [240,241,243,244].

#### 5.2.2. Broccoli Sulforaphane

Cruciferous vegetables, such as cauliflower, broccoli sprouts, kale, and cabbage, are rich in the bioactive compound sulforaphane. They have become a popular topic in cancer prevention in recent years. Sulforaphane is widely known for its chemopreventive properties through its ability to activate detoxification enzymes in the liver and enhance the body’s ability to eliminate carcinogens and toxins [245]. Some anticancer effects of sulforaphane include inducing apoptosis and cell cycle arrest, inhibiting angiogenesis and invasion, diminishing inflammation, and including cellular defenses as a potent Nrf2 inducer [246,247].

The effectiveness of sulforaphane in combating BC has been well documented. An in vitro study found that sulforaphane can reduce the potential of BC stem cells [248]. Other preclinical studies found that sulforaphane inhibits cell growth among ER+ and PR+ MCF-7 cells due to its anti-proliferative and anti-invasive effects [249,250,251]. In addition, sulforaphane treatment significantly reduced TNBC tumor size and inhibited the renewal of stem cells in mouse models [252]. This study also showed that implementing supplementation before the onset of the tumor had a stronger effect on reducing the tumor volume compared to the sulforaphane-post-treated group, suggesting a strong preventive effect of sulforaphane [252]. The effectiveness of sulforaphane appears to be influenced by its dosage. At a concentration of 40 µM, sulforaphane promoted apoptosis and necrosis in a TNBC cell line, MDA-MB-231. Lower doses of sulforaphane ranging from 20 to 40 μM of sulforaphane led to the down-regulation of genes associated with the epithelial–mesenchymal transition, a biological process of cancer metastasis [253]. Additionally, sulforaphane is recognized for its roles in regulating epigenetic pathways. Specifically, it targets HDAC and DNA methyltransferases (DNMTs), leading to modifications in gene transcription and expression in cancer cells [248,254]. Our studies proved that dietary broccoli sprout consumption during the maternal stage may affect tumorigenesis in the offspring by regulating the epigenetic processes [255]. 

Sulforaphane can be applied as a potential adjuvant chemotherapy. Combining 5-fluorouracil with sulforaphane increased cell autophagy, leading to decreased cell growth and increased apoptosis in MDA-MB-231 cells [256]. Sulforaphane can facilitate the mitigation of the cardiotoxicity of doxorubicin (DOX) by improving mitochondrial integrity in HER2+ BC [257]. Furthermore, the combination of sulforaphane and genistein also showed a stronger protective effect on BC tumorigenesis by increasing the rate of apoptosis and down-regulating KLF4 and HDAC compared to any treatment alone [258]. In a clinical trial, a group of women with abnormal mammograms was randomized to receive a broccoli glucoraphanin, the precursor of sulforaphane. The result showed that the supplementation was linked to decreased HDAC activity, and significant changes were observed in Ki-67 and HDAC3 levels before and after the treatment [259]. Moreover, a group of women undergoing reduction mammoplasty consumed a broccoli sprout preparation containing 200 µmol of sulforaphane prior to surgery. The results demonstrated that sulforaphane from broccoli sprouts reaches and exerts pharmacodynamic effects in human breast tissue. Sulforaphane metabolites were easily detectable in human breast tissue that was enriched with epithelial cells [260]. The findings support further investigation into the potential chemopreventive role of sulforaphane, particularly in BC.

Broccoli sulforaphane intake can affect the gut microbial distribution and composition. In a study comparing a glucoraphanin-treated high-fat diet to a control diet in mice, it was observed that glucoraphanin led to a reduction in the relative abundance of the *Desulfovibrionaceae* family [261]. This family is recognized for its potential to produce endotoxins and is positively associated with plasma lipopolysaccharides [262]. One in vivo study found significant differences in fecal microbiota composition between the sulforaphane group and the control group. Specifically, there was a notable increase in the abundance of *Lactobacillus* in the treated group [261]. Research has shown that *Lactobacillus* exhibits anti-tumor activities both in laboratory settings and in animal models of BC [263]. Moreover, *Lactobacillus* bacteria have been found to produce aryl hydrocarbon receptor (AHR) ligands, including tryptophan metabolites. The AHR signaling pathway is crucial in regulating immune responses and inflammation. Thus, the potential of sulforaphane to ameliorate BC may involve modulation of the AHR signaling pathway through microbial tryptophan metabolism, highlighting the intricate interplay between dietary compounds, gut microbiota, and host physiology in cancer prevention and treatment [264]. Table 3 presents additional insights into the relationship between broccoli sulforaphane and gut bacteria, highlighting key findings such as reductions in harmful bacteria like *Desulfovibrio* and increases in beneficial species such as *Lactobacillus*, with potential implications for cancer prevention in response to sulforaphane treatment [261,265,266].

#### 5.2.3. Green Tea Polyphenol

Over the past few decades, green tea has been studied for its preventive effect on carcinogenesis. Tea is rich in polyphenols that exhibit strong anti-inflammatory, antioxidant, and antimutagenic characteristics across diverse biological systems [267]. The major components of green tea include epicatechin (EC), epigallocatechin (EGC), epicatechin-3-gallate (ECG), and epigallocatechin-3-gallate (EGCG) [268]. Among all these polyphenols, EGCG is considered the most effective and abundant polyphenol in green tea, accounting for almost 50% to 75% of the total catechins [268]. Tea polyphenols are potent antioxidants against excess free radicals, thereby altering signaling pathways and preventing oxidative damage to lipids, proteins, and DNA [267,269].

Meta-analyses focused on the relationship between the consumption of green tea and BC risk and found an inverse association between the high consumption of green tea and the risk of BC recurrence [270,271,272]. Further in vitro studies showed that the treatment of green tea polyphenolic extracts can suppress BC proliferation through cell cycle arrest [273]. Moreover, tea polyphenols exerted apoptotic effects on BC cells by down-regulating the activity of telomerase and survivin expression [274,275]. It also has an antiangiogenic effect by inhibiting vascular endothelial growth factor (VEGF) transcription [276]. Animal studies indicate that the green tea component may potentially increase BC latency and decrease tumor weight and the risk of metastasis [277,278]. In addition, the combinatorial treatment of green tea and broccoli extracts exerted the strongest effect on suppressing tumor volume compared to any treatment alone [278]. Green tea treatment can also enhance the treatment efficacy of tamoxifen by reducing the tumor size and promoting apoptosis in a resistant animal model [279]. A clinical trial study in women with primary BC showed that women with either benign or malignant cells had a reduced level of proliferation biomarker, ki-67, after tea consumption compared to the control group [280].

Green tea polyphenols may improve gut dysbiosis and reduce the pro-inflammatory substances [281]. They can inhibit the proliferation of pathogens in the gut community and support the growth of beneficial bacteria like *Bifidobacterium*, which improves the gut environment [73]. A study indicates that either broccoli sprouts or green tea consumption led to different gut microbiota clustering and higher beta diversity compared to the control; for example, increased levels of the genera *Allobaculum*, *Lactobacillus*, and Lachnospiraceae and a significant decrease in *Lactococcus* and *Proteobacteria* [278]. Similarly, the in vitro study suggests that tea compounds increased *Bifidobacterium* spp., *Lactobacillus*, and *Enterococcus* [281]. Tea polyphenols can inhibit harmful bacteria like *Bacillus cereus*, *Campylobacter jejuni*, *Clostridium perfringens*, *E. coli*, and *Helicobacter pylori*, while supporting the growth of beneficial bacteria such as *Bifidobacterium* spp., *Lactobacillus*, and *Enterococcus* [282]. Tea polyphenols can also increase the enrichment of SCFA-producing bacteria leading to the increased production of SCFAs [283,284,285,286]. 

#### 5.2.4. Curcumin

Curcumin, a natural compound derived from turmeric, has been widely known for its antioxidant, anticancer, and chemopreventive properties via complicated intracellular signaling pathways [287]. Curcumin is potent in modulating the expression and function of proteins such as inflammatory cytokines and transcription factors associated with cell proliferation [288,289]. Specifically, curcumin inhibits the signaling of NF-κB, signal transducers and activators of transcription (STAT)-3, β-catenin, peroxisome proliferator-activated receptor (PPAR)-γ, and notch-1 [288]. Therefore, curcumin acts as an anticancer agent against multiple cancer types [290]. Curcumin can also suppress the zeste homolog 2 (EZH2) gene, an up-regulated epigenetic factor responsible for BC proliferation, leading to the restoration of the tumor suppressor gene, and therefore inhibiting the invasion of cancer cells [291]. In addition, curcumin shows anti-angiogenesis properties by regulating pro-angiogenesis factors such as VEGF [292]. 

Curcumin can effectively reduce the proliferation in both ER+ and ER- BC cells. It acts as a phytoestrogen to inhibit the growth of BC cells by competing with the endogenous estrogen. In addition, one study showed the combinatorial effects of curcumin and its analogs on inhibiting hormone factor HER2 by inhibiting the HER2 tyrosine kinase [293]. Some studies validated that the treatment of curcumin is associated with a reduced expression of ERα and p53, resulting in effectively suppressing BC cell proliferation and invasion [294,295]. Considering the metastasis spread to the lungs, livers, and bones in the case of advanced BC, curcumin shows protective effects against this invasion [296]. In addition, studies have shown that curcumin can significantly reduce tumor weight and volume in multiple BC mouse models [297,298]. Similar to other bioactive compounds, curcumin enhances the treatment efficacy of conventional chemotherapies such as Paclitaxel, Cisplatin, and Doxorubicin [299]. Curcumin increased the efficacy of Paclitaxel by down-regulating PI3K and increasing the apoptotic rate compared to the Paclitaxel treatment alone [300]. Human studies also demonstrated an improved response rate and physical performance in the group treated with curcumin and paclitaxel compared to the placebo [301]. Although many preclinical studies support the anti-tumor effects of curcumin in BC by targeting various signaling pathways, the clinical evidence supporting the use of curcumin in BC treatment is limited.

Some studies proposed that curcumin may exert direct effects on the gut, as evidenced by altered gut microbiome composition and function. Also, curcumin may generate beneficial metabolites after the transformation occurs in the gut [302]. One study found that curcumin led to alterations in urine metabolites that are associated with energy production, fatty acids, and anti-inflammation pathways [303]. Curcumin promotes the growth and proliferation of the beneficial gut bacteria *Bifidobacteria* and *Lactobacilli* and butyrate-producing bacteria and reduces the production of pathogenic ones that are associated with systemic diseases [304]. Curcumin reduces the abundance of *Coriobacterales*, *Prevotellaceae*, *Enterococci*, and *Enterobacteria*, implicating modulation in immune function [305]. Specific strains like *Lactobacili* may have inhibitory effects on pathogens and prevent the invasion of human epithelial cells [306]. The modulation also results in a favorable shift characterized by a reduction in *Dorea* and the *Firmicutes*/*Bacteroidetes* ratio, which is associated with metabolic health [307]. In the human placebo-controlled trial, curcumin and turmeric treatment led to distinct gut microbiota variations. Curcumin supplements increased the gut bacteria species while the placebo group showed reduced species [308]. One interesting aspect is the mutual interaction between the gut microbiota and curcumin. The gut bacteria can metabolize curcumin and generate new metabolites, such as tetrahydrocurcumin, dihydrocurcumin, and ferulic acid, which may exhibit anticancer effects [309,310].

#### 5.2.5. Resveratrol

Resveratrol, a non-flavonoid polyphenol, is naturally present in grapes, berries, red wine, and peanuts. It exists in two main forms: cis-resveratrol and trans-resveratrol. Trans-resveratrol, in particular, has garnered significant attention for its anti-tumor, antioxidant, and anti-inflammation properties in many cell cultures and animal models [311]. For dietary supplements, resveratrol is commonly extracted from Fallopia japonica (Japanese knotweed), a plant known for its high resveratrol content. Many studies have demonstrated that resveratrol has a chemopreventive effect against the development of lung, skin, breast, and prostate cancer [312]. Specifically, resveratrol affects each stage of cancer progression by modulating the signaling pathways associated with inflammation, angiogenesis, and metastasis [313]. It shows inhibitory effects on tumor activity by altering cyclin, leading to p53-dependent cell cycle arrest at the G0/G1 phase [314]. 

In relation to BC, resveratrol inhibits the growth of MCF-7 BC cells via the modulation of cyclin expression [315,316]. Also, its antioxidant properties prevent tumor initiation by suppressing the free radicals, mitigating lipid peroxidation, and protecting against DNA damage [317]. Similar to curcumin, resveratrol exhibits anti-inflammatory effects by targeting the NF-κB pathway, resulting in the suppression of inflammatory mediators and inhibition of apoptosis-promoting factors [318]. Inflammation may create an environment favorable for the formation and development of tumors because pro-inflammatory factors are associated with tumor microenvironment [319]. A preclinical study showed that resveratrol can reduce the activity of cyclooxygenase (COX)-1 and -2 enzymes, resulting in a decrease in the production of prostaglandin E2 (PGE2) [320]. 

Among BC patients, one study found that the resveratrol treatment significantly decreased PGE2 expression in nipple aspirate fluid, associated with decreased methylation of the tumor suppressor gene *RASSF-1a* [321]. In an animal model, resveratrol suppressed NF-κB DNA-binding activity and inhibited mammary tumorigenesis. In addition, it prevented the growth of BC cells in a dose-dependent manner [322]. Another animal study showed the inhibitory effects of a combination treatment of resveratrol and 17β-estradiol on estrogen-induced BC by up-regulating Nrf2 in breast tissues [323]. Similar results on inhibiting BC progression were reported in these animal studies [324,325,326,327]. A case–control study revealed that women who consumed high levels of resveratrol had a relative low BC risk compared to women with low resveratrol intake [328]. Resveratrol also exerts synergistic effects by sensitizing BC cells to cell death when combined with other anticancer drugs such as Paclitaxel [329]. 

Many studies suggested that the health benefits of resveratrol may be attributed to its effects on modulating the gut microbiome and protecting the intestinal barrier. Considering the low bioavailability of resveratrol and the derived metabolites that interact with the microbiome in the gut, it is important to understand the interactions between resveratrol and the gut microbiome in cancer treatment and the anticancer properties of these metabolites [330,331]. Studies have shown that *Slackia equolifaciens* and *Adlercreutzia equolifaciens* can convert resveratrol to bioactive dihydroresveratrol by generating the hydrogenation of the double bond. Resveratrol can also be converted to different metabolites such as 3,4-dihydroxy-trans-stilbene and 3,4-dihydroxybibenzyl (lunularin) by the gut microbiome, which were identified in both in vivo and in vitro studies and demonstrated anti-inflammatory properties [332]. 

Among all the resveratrol-derived metabolites, dihydroresveratrol and lunularin are most abundant in tissues and gut than resveratrol. In an animal model, dihydroresveratrol and lunularin showed anticancer and anti-proliferation properties and protected renal and colonic cell lines [333]. However, not many studies have focused on the interactions between BC cells and resveratrol metabolites modulated by the gut microbiome. In addition, resveratrol has an antifungal effect and limits the invasion of harmful bacteria. It reduces the abundance of the bacteria *Enterococcus*, *Escherichia coli*, and *Clostridium* but increases the *Bacteroidetes*-to-*Firmicutes* ratio and benefits the growth of *Bacteroides*, *Lactobacillus*, and *Bifidobacterium*, leading to the protection of the intestinal barrier [334,335,336]. Both *Enterococcus* and *Escherichia coli* contribute to the ROS, suggesting that the antioxidant properties of resveratrol and protection against intestinal damage are modulated by the gut microbiome [337]. Similar study results highlighting the potential impacts of resveratrol on gut health and metabolic balance are presented in Table 3 [338,339].

**Table 3:** Phytochemicals and their connection with BC prevention and bacterial regulation.

Phytochemical	Connection with Bacterial Regulation	Connection with BC Prevention
Soy isoflavone	↑ *Lactobacilli*, *Bifidobacteria*, and *Firmicutes/Bacteroidetes* ratio [241] ↑ *Lachnospiraceae*, *Ruminococcaceae* [240] ↑ *Lactobacillus*, *Bifidobacterium*, and *Bacteroides* ↓ *Clostridium* [243] ↑ Microbiome diversity ↑ *Enterococcus* ↓ *Lactobacilli* and *Ruminococcus* [244]	Suppresses BC cell growth and induces programmed cell death by blocking the function of various enzymes [224] Enhances antioxidant protection and DNA [224] Inhibits NF-κB activation and controls PI3K/Akt or MAPK/ERK [224]
Sulforaphane	↓ *Desulfovibrio* ↑ *Lactobacillus*, correlating with decreased methylation capacity and enhanced anti-tumor tryptophan metabolites [261] ↑ *B. fragilis* and *Clostridium cluster I* [265] ↑ *Bacteroidetes/Firmicutes* ratio, *Proteobacteria*, *Akkermansia muciniphila* ↓ *Mucispirillum schaedleri* [266]	Exerts anti-proliferative effects by modulating purine and amino acid metabolism while also demonstrating antioxidative properties [247] Elevates sulfate and glutathione-related metabolites and reduces global DNA methylation in tumor tissue [261]
Green tea polyphenol	↓ *Bacillus cereus*, *Campylobacter jejuni*, *Clostridium perfringens*, *E. coli*, *Helicobacter pylori*, *Legionella pneumophila*, and *Mycobacterium* spp. [282] ↑ *Bifidobacterium* spp., *Lactobacillus*, and *Enterococcus* ↑ SCFAs [283] ↓ *Bacteroides*, *Prevotella*, *Clostridium hystoliticum*, *Eubacterium*, and *Clostridium* [284] ↑ *Akkermansia* ↓ *Firmicutes* and alpha diversity [285] ↑ SCFA-producing bacteria (*Faecalibacterium*, *Roseburia*, *Blautia*, *Eubacterium*, *Bifidobacterium*, and *Coprococcus*) [286]	Induces cell cycle arrest; inhibits cell growth and induces apoptosis by inhibiting survivin expression [275] Exerts an antiangiogenic effect by inhibiting VEGF transcription [276]
Curcumin	↑ *Bifidobacteria*, *Lactobacilli*, and butyrate-producing bacteria ↓ *Prevotellaceae*, *Coriobacterales*, *Enterobacteria*, and *Rikenellaceae* [304] ↓ *Dorea* and *Firmicutes/Bacteroidetes* ratio ↑ *Butyricicoccus*, *Stomatobaculum*, *Acetatifactor*, *and Vampirovibrio* [307]	Inhibits invasion and BC-cell proliferation by modulating protein kinase and transcription factors, and promotes apoptosis by producing ROS [289,340] Inhibits NF-κB, STAT-3, β-catenin, PPAR-γ, and notch-1 signaling pathways [340] Exhibits anti-angiogenic properties [340] Improves the function of the intestinal barrier and relieves inflammation and oxidative stress in the gut [338]
Resveratrol	↓ *Firmicutes* ↑ *Bacteroidetes*, *Alistipes*, *Rikenella*, *Odoribacter*, *Parabacteroides*, and *Alloprevotella* [338] ↑ *Bacteroides*, *Lachnospiraceae_NK4A136_group*, *Blautia*, *Lachnoclostridium*, *Parabacteroides*, and *Ruminiclostridium_9* [339]	Inhibits tumor growth and increases latency by inducing apoptosis and inhibiting 17β-estradiol-mediated increase in DNA damage in mammary tissues [323] Improves the function of the intestinal barrier and relieves inflammation [339] Regulates lipid metabolism and prevents weight gain [339]

↑ Increased changes; ↓ Decreased changes

## 6. Nutrition-Based Microbial Treatment in BC

Recent research indicates a connection between gut dysbiosis in both the breast and gut communities and the development and progression of BC. BC pathogenesis is often associated with ongoing inflammation triggered by intestinal bacteria-activated signaling pathways [341]. Traditional BC treatments include surgery, chemotherapy, radiation, endocrine therapy, and targeted therapy. However, there are always concerns about side effects on normal cells and induced drug resistance during conventional cancer treatment [342]. Hence, exploring novel treatment strategies featuring higher therapeutic efficacy and fewer complications has become a popular topic in cancer prevention and therapy. Nutrition and microbial therapy, including the use of probiotics and prebiotics, are among the hottest topics in the field.

Probiotics are beneficial microorganisms that benefit the host when consumed in adequate amounts. They are known for producing antibiotics and anti-carcinogens. Probiotics are commonly found in fermented foods and dietary supplements [15]. Probiotics offer well-established benefits, including preserving gut microbiome community structure, enhancing mucosal barrier function, and protecting against pathogens.

Prebiotics are a type of nondigestible fiber that promotes the growth of beneficial bacteria in the intestinal tract. Common sources of prebiotics include certain fruits, vegetables, and whole grains. Consuming prebiotic-rich foods can help support a healthy balance of gut bacteria, which in turn can benefit digestion, immune function, and overall health. For example, *Bacteroidetes* and *Firmicutes* phyla convert these nondigestible fibers into phytoestrogens and SCFAs, which exhibit properties that suppress tumor growth and have anti-estrogenic and anti-proliferative effects. Dietary fiber has the potential to modify the composition of the gut microbiota, which in turn can impact the metabolism of estradiol. This influence occurs via the modulation of specific enzyme activities, such as β-glucuronidase [343]. 

Given the association between gut dysbiosis and tumorigenesis, probiotics play a crucial role in restoring balance to the gut microbiome and promoting overall homeostasis [344,345]. Probiotics can effectively inhibit cancer initiation, progression, and metastasis by beneficially modulating the composition and activity of the gut microbiome, such as stimulating the growth of beneficial bacteria groups such as *Bifidobacterium* and *Lactobacillus* [346,347]. Research findings have demonstrated that certain probiotic strains can regulate the gastrointestinal microbiome and immune responses. These probiotics can be utilized as a preventative measure against cancer and supplementary therapy during chemotherapy [348]. 

Several in vitro and in vivo studies show that probiotics like *Bifidobacterium* and *Lactobacillus* can inhibit cancer cell proliferation and induce cancer cell apoptosis [349]. The potential mechanisms underlying probiotic-induced anticancer effects include alterations in gut microbial composition and metabolic activity, the production of anticancer substances like SCFAs, and enhancing intestinal barrier integrity [350]. SCFAs, especially butyrate, are microbially produced metabolites that control the growth of pathogens and inhibit the invasion of cancer cells by limiting HDAC activity and the modulation of gene expression [351]. In the context of BC, including TNBC, SCFAs have been used for synergistic anticancer treatment and other cancer treatment drugs to reduce drug resistance [352]. In an animal model with breast tumors, the treatment group with oral supplementation of *Lactobacillus acidophilus* showed a longer survival time than the control group, which may be due to the production of pro-inflammatory cytokines like IFN-γ stimulated by the probiotics [74]. Similarly, the oral administration of *Lactobacillus plantarum* and selenium nanoparticles in BC-bearing mice reduced tumor weight and increased the survival rate by increasing the pro-inflammatory cytokines IFN-γ, TNF-α, and IL-2 levels and NK cell activity [76]. 

Although a number of preclinical studies provide solid evidence about the efficacy of probiotics in inhibiting BC, the relevant clinical evidence is limited, especially in the measurement of BC outcomes. Nevertheless, two clinical studies supported the role of probiotics in alleviating chemotherapy-induced complications, including weight gain, metabolic changes, and increased plasma low-density lipoprotein [353,354]. Future studies focusing on determining the optimal strains and dosage of probiotics will help the development of a novel therapeutic approach tailored to the clinical features of BC. 

Despite the direct administration of probiotics, fermented food products enriched in lactic acid bacteria are also related to a reduced risk of BC in several epidemiological studies [355]. Many fermented vegetables such as cabbage, olives, sauerkraut, kimchi, and cucumbers, along with fermented dairy products like yogurt, kefir, and cheese as well as meat products such as salamis and sausages, serve as rich sources of fermentation bacteria [145]. Milk fermented by lactic acid bacteria and several *Bifidobacteria* strains demonstrated anti-proliferative effects on MCF-7 BC cell lines [356]. Similarly, a case–control study showed that lower consumption of fermented milk is related to a higher BC incidence [357]. Consistently, another study revealed that high non-fermented milk consumption in the long term was related to an increased incidence of ER+/PR+ BC [358]. Also, consuming fermented soy products relates to a significant decrease in the risk of nonlocalized BC [359]. In preclinical settings, mice consuming fermented milk with *Lactobacillus helveticus* had a lower risk of developing BC malignancy [360]. Those mice with metastatic BC had a better prognosis after the oral administration of *Lactobacillus brevis* [361]. Moreover, mice injected with BC cells were treated with kefir water containing various beneficial probiotics and showed reduced tumor size and weight with an enhanced immune response [362]. 

Fecal microbiota transplantation (FMT) has been recognized as a novel way to restore the gut microbiome and reverse dysbiosis for cancer patients without interrupting the natural balance of the gut microbiome [363]. FMT procedures have been widely used to treat certain GI conditions such as *clostridioides difficile* infection (CDI) and inflammatory bowel diseases (IBDs) by transferring the microbiome from healthy populations to the patients. Several animal studies support the anti-tumor activities of FMT. Among irradiated mice, FMT alleviates the GI syndrome triggered by radiation by enhancing the intestinal barrier and restoring the beneficial bacteria. Also, FMT helps maintain the genetic activity and regulatory functions in the small intestine [364]. Moreover, due to the fact that the gut microbiome plays an important role in improving the efficacy and relieving the toxicity of chemotherapy, FMT can effectively enhance GI cell recovery from chemotherapy by remodulating the gut microbiome [365]. Immunotherapy has shown remarkable progress in recent years, with the emergence of ICIs like anti-PD-1/PD-L1 and anti-CTLA-4. Preclinical studies using mouse models have shown that FMT has the potential to affect the responsiveness of patients with malignant tumors to ICIs by changing the tumor microenvironment and boosting the immunogenicity of the tumor [366]. However, the large-scale clinical studies involving FMT are limited, and research on FMT in the context of BC is still in its early stages. 

In addition to probiotics, FMT, and fermented diets, a combination treatment of bioactive compounds and microbial treatment for BC offers promising avenues for BC therapy. Bioactive compounds like soy isoflavone, sulforaphane, green tea polyphenols, curcumin, and resveratrol exhibit anti-inflammatory and antioxidant properties that may impact BC progression. Incorporating these compounds into microbial treatment strategies offers a comprehensive approach to tackling BC, which could lead to improved treatment outcomes and patient well-being. One animal study found that the combination treatment of *Lactobacillus* and soy milk effectively reduced the incidence, multiplicity, and volume of mammary tumors [367]. A clinical trial in Japanese BC patients found that consuming probiotic beverages with *L. casei* Shirota and soy isoflavones was associated with a lower risk of BC [368]. However, another clinical trial investigated the combinatorial effect of soy and probiotic supplementation and found that such treatment did not affect the hormone levels in postmenopausal women with or without BC [369]. In other two clinical trials, soy milk fermented with *Lactobacillus* enhanced the bioavailability of isoflavones, particularly genistein, compared to soy milk alone [370,371]. This indicates that probiotics can potentially alter the metabolism of isoflavones from soybean products, consistent with the evidence showing reduced excretion of genistein and daidzein in urine [370]. Conversely, another clinical trial showed that regular soy intake did not significantly affect plasma or urinary isoflavones, but probiotic yogurt or resistant starch (prebiotic) intake led to a slight increase in circulating levels of daidzein and genistein [372]. The combinatorial effect of soy and probiotics on BC treatment remains an area with limited research. Further research is needed to explore the potential therapeutic role and underlying mechanisms of combining dietary compounds and probiotics in BC management.

**Figure 1:**
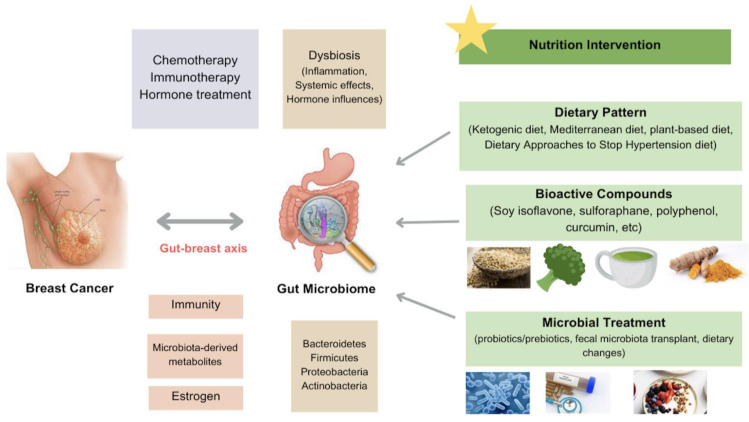
A schematic graph depicts the mechanistic interaction between nutrition/food, the gut microbiome, and BC.

Delving deeper into the intricate mechanisms and interactions underlying these interventions is imperative for maximizing their potential in managing BC effectively. Figure 1 emphasizes the crucial role of nutrition interventions on the gut–breast axis and the interactions with biological factors and potential mechanisms. Gut dysbiosis causes the dysregulation of metabolites, impacting many physiological processes associated with BC initiation and development. Microbes inhabiting the gut have been associated with various aspects of inflammation, immune responses, genetic expression, hormonal pathways, and the metabolism and effects of drugs [373]. Modulating the gut microbiota through nutrition interventions such as dietary patterns, bioactive compounds, fermented foods, probiotics/prebiotics, and FMT could be a promising adjuvant therapeutic strategy for BC treatment. These interventions may help restore a healthier gut microbiota composition, which in turn could positively influence BC outcomes by mitigating inflammation, enhancing immune responses against tumor cells, regulating hormonal balance, and optimizing the efficacy of anticancer drugs. Understanding the intricate interplay between nutrition, gut microbiota, and BC pathogenesis is essential for developing personalized and effective strategies for BC prevention and treatment.

## 7. Conclusions

Nutrition intervention and microbiome modulation hold significant promise in the management of BC. The microbiota plays a crucial role in maintaining overall human health, and disruptions in its balance can lead to pathobiological changes, including BC. While the correlation between the microbiome and BC is well established, significant questions remain regarding the intricate interactions between nutrition, the microbiome and BC. Addressing these questions will require large-scale studies, including animal models and clinical trials. Additionally, exploring bioactive compounds in foods, such as polyphenols, isoflavones, and other phytochemicals may offer novel strategies for modulating the microbiome and potentially influencing BC progression. Incorporating these bioactive compounds into personalized dietary interventions could provide innovative approaches to BC management and prevention. Finally, the advancement of engineered bacteria, such as those found in probiotic products, along with the development of functional foods that promote a healthy balance of gut bacteria (eubiosis), represents an innovative approach in the field of BC clinical practice.
